# Prediction of bitterness based on modular designed graph neural network

**DOI:** 10.1093/bioadv/vbae041

**Published:** 2024-03-13

**Authors:** Yi He, Kaifeng Liu, Yuyang Liu, Weiwei Han

**Affiliations:** Key Laboratory for Molecular Enzymology and Engineering of Ministry of Education, School of Life Science, Jilin University, Changchun 130012, China; Key Laboratory for Molecular Enzymology and Engineering of Ministry of Education, School of Life Science, Jilin University, Changchun 130012, China; Key Laboratory for Molecular Enzymology and Engineering of Ministry of Education, School of Life Science, Jilin University, Changchun 130012, China; Key Laboratory for Molecular Enzymology and Engineering of Ministry of Education, School of Life Science, Jilin University, Changchun 130012, China

## Abstract

**Motivation:**

Bitterness plays a pivotal role in our ability to identify and evade harmful substances in food. As one of the five tastes, it constitutes a critical component of our sensory experiences. However, the reliance on human tasting for discerning flavors presents cost challenges, rendering in silico prediction of bitterness a more practical alternative.

**Results:**

In this study, we introduce the use of Graph Neural Networks (GNNs) in bitterness prediction, superseding traditional machine learning techniques. We developed an advanced model, a Hybrid Graph Neural Network (HGNN), surpassing conventional GNNs according to tests on public datasets. Using HGNN and three other GNNs, we designed BitterGNNs, a bitterness predictor that achieved an AUC value of 0.87 in both external bitter/non-bitter and bitter/sweet evaluations, outperforming the acclaimed RDKFP-MLP predictor with AUC values of 0.86 and 0.85. We further created a bitterness prediction website and database, TastePD (https://www.tastepd.com/). The BitterGNNs predictor, built on GNNs, offers accurate bitterness predictions, enhancing the efficacy of bitterness prediction, aiding advanced food testing methodology development, and deepening our understanding of bitterness origins.

**Availability and implementation:**

TastePD can be available at https://www.tastepd.com, all codes are at https://github.com/heyigacu/BitterGNN.

## 1 Introduction

Humans can perceive five major distinct tastes: sweet, bitter, sour, salty, and umami (Witt 2019). Bitterness is often associated with recognizing and preventing the consumption of toxic foods (Wooding *et al.* 2021). However, not all bitter tastes are harmful; some bitter compounds promote health, as seen in vegetables (Drewnowski and Gomez-Carneros 2000) and clinical drugs (Mennella *et al.* 2013), promoting appetite, increasing food flavor, and preventing overconsumption. Therefore, determining whether a compound is bitter is crucial in the screening process for sweeteners and bitterants. Since humans cannot taste all studied compounds, predicting bitterness using computational methods is particularly important.

In silico approaches for predicting bitterants are primarily divided into structure-based and ligand-based methods. However, the diversity of physicochemical properties and structural characteristics of the molecules studied, along with the complexity and ambiguity of receptor structures, make predicting bitterness challenging. One solution to this problem involves traditional machine learning (ML) techniques, including AdaBoost (AB) (Freund and Schapire 1997), support vector machines (SVM), and random forests (RF). BittersweetForest, a random forest model based on molecular fingerprints, achieved a 95% accuracy and 0.98 AUC in a cross-validation study (Banerjee and Preissner 2018). BitterX, which utilizes the SVM method, demonstrates good performance in predicting bitterness according to chemical descriptors (Huang *et al.* 2016), but the dataset for BitterX training was small and its website is not suitable for large-scale predicting. Dagan-Wiener et al combined physical and chemical descriptors with an “AdaBoost-based” ML classifier for bitter prediction (Dagan-Wiener *et al.* 2017), but many of the descriptors do not provide useful information that helps to interpret structural features. Another solution to this problem is deep learning (DL) approaches. Bo et al. conducted bitterness prediction using a multi-layer perceptron (MLP) based on molecular descriptors and molecular fingerprints, respectively (Bo *et al.* 2022). Additionally, a method was proposed to convert molecules into images and classify them as bitterants or not using a convolutional neural network (CNN). Fingerprint-based MLP performed the best but they didn't provide the code. Bitter peptide prediction using natural language processing (NLP) is sequence-based and not suitable for structure-based prediction for small molecules (Charoenkwan *et al.* 2021).

Moreover, most deep learning flavor prediction work focuses on sweetener prediction, making it necessary to develop deep learning models for bitterant prediction. Graph neural networks are gaining popularity for molecular property prediction and classification. GCN (Kipf and Welling 2017), GAT (Veličković 2023), GATv2 (Brody *et al.* 2022), were initially applied to node classification of graphs, without considering edge information. GCN assumes that all nodes contribute equally to the representation of a node, which may not always be the case. These methods can be simply transformed into entire graph classification, such as using DGLife (https://lifesci.dgl.ai/index.html). Molecular classification based on graph convolutional networks is a full graph classification problem, with nodes as atoms and edges as chemical bonds. Graphs and molecules seem to have good natural adaptability in structure. NFP first applied graph convolutional networks to molecular fingerprints, did not require fixed-size molecular input, and simply aggregated the information of edges and nodes (Duvenaud *et al.* 2015). Weave employed a hybrid learning approach for edges and nodes, guaranteeing isomorphism and proposing a molecular layer representation with less information loss in the readout function (Kearnes *et al.* 2016). Later, neural fingerprints made significant progress in predicting quantum chemical properties of molecules, such as SchNet (Schütt *et al.* 2017), MPNN (Gilmer *et al.* 2017), MGCN (Lu *et al.* 2019). To improve graph neural networks, GIN (Xu *et al.* 2019, Hu *et al.* 2020a) achieved a new embedded learning approach, OGB (Hu *et al.* 2020b) virtualized nodes, and PAGTN\(Chen *et al.* 2019) proposed new attention mechanisms. It was later suggested that AFP could more effectively capture atom information than Weave and NFP (Xiong *et al.* 2020). GraphSAGE (Hamilton Ying and Leskovec 2017) can generate node embeddings for previously unseen data, making it capable of handling dynamic graphs. It also uses a sampling strategy to control the number of neighbors, which helps to scale to large graphs. The sampling strategy can cause information loss and may not work well on graphs where a node’s neighborhood is essential for its representation. There are also proposals for 3D fingerprint learning (Wang *et al.* 2020), but complex inputs may increase computational costs. It is worth considering whether combining multiple methods could result in a better graph neural network.

In this study, we propose a new graph neural network generation strategy and identify an excellent GNN model HGNN. Using HGNN and other well-performed GNNs, we built outstanding bitterness predictors and systematically analyzed the properties of bitterants. This work provides a method reference for graph neural network design and molecular taste recognition based on deep learning.

## 2 Methods

### 2.1 Flow chart

**Figure 1.**
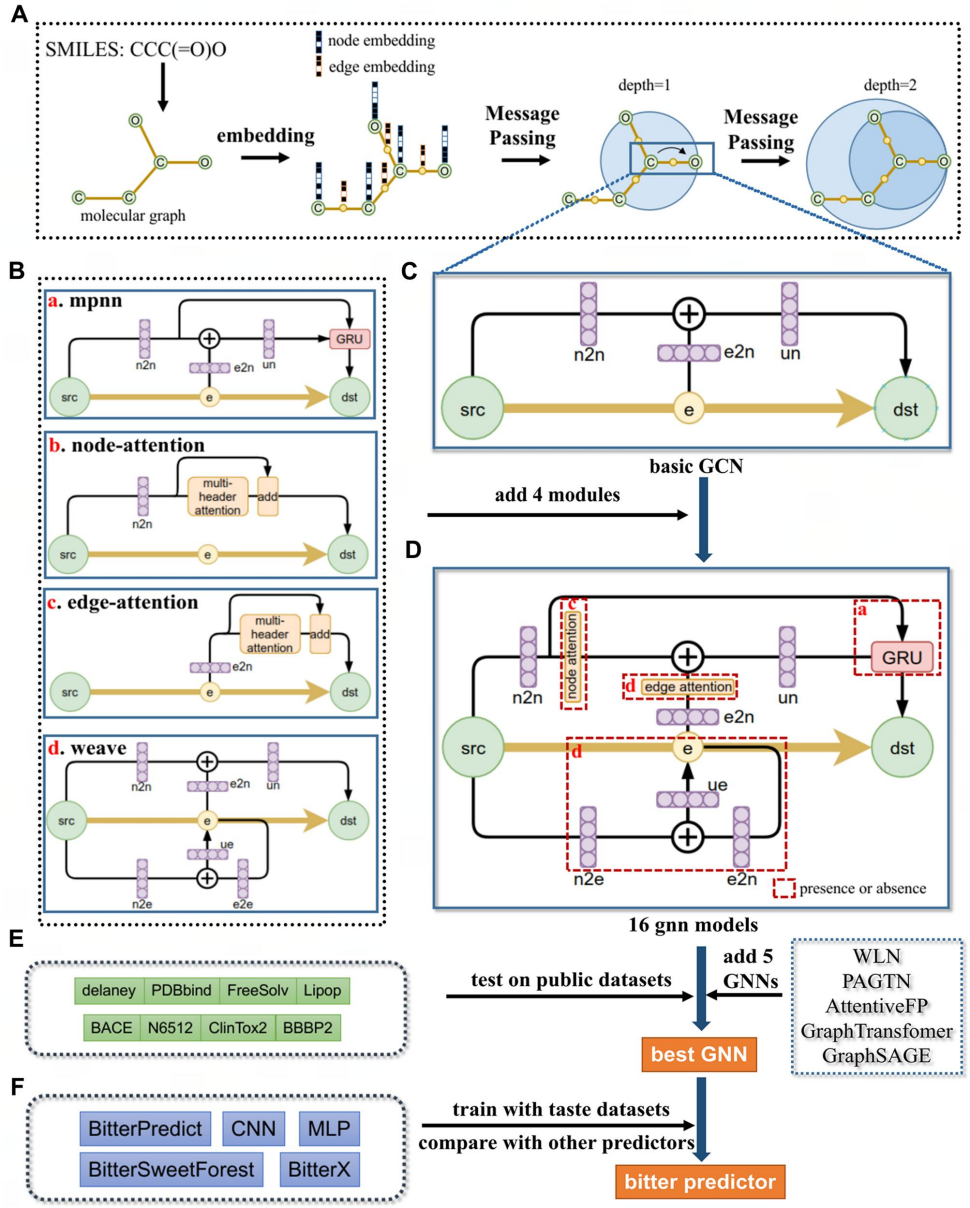
Chart of the study. (A) The process of transmitting information in the molecular graph. (B) The basic 4 GNN modules, it shows how the embedding feature of source node (src) transfer to node of destination (dst), the linear layer is colored with purple, e2n represents edge to node, and in the same way, n2n, n2e, e2e represents node to node, node to edge, edge to edge, respectively. (C) Basic GCN framework. (D) Add 4 GNN modules to the GCN framework, a, b, c and d correspond to the 4 modules in Figure 1 B, each module can be absent and present, so there will be 16 combined models. (E) 8 Public datasets. (F) Existing models for bitterness prediction.

Our workflow can be shown in Fig. 1. First we design the GNN module with edge attention, and then merge it into the basic GCN architecture (Fig. 1C) with three other traditional GNN models GAT, weave and MPNN (Fig. 1B) to obtain a GNN containing four basic GNN modules (Fig. 1D), each of modules has a state of presence or absence and a fixed position, thus generating 16 (2 × 2 × 2 × 2) son models. The performance of the 16 son models and other 5 popular GNNs are compared on the public datasets (Fig. 1E), and the best 4 GNN are selected as the deep learning model for bitter prediction. The trained bitterness predictors are called BitterGNNs and will compare its performance with other existing bitterness predictors (Fig. 1F).

### 2.2 Modular design of GNN

The structure of the modular design can be seen in Fig 1 BCD. The algorithms of GATv2 (Veličković 2023), MPNN (Schütt *et al.* 2017), and weave (Duvenaud *et al.* 2015) have been given in their corresponding articles, and here we give the algorithm for edge attention:

(1) $$\mathrm{es}(h_{i},e_{j})=\mathrm{LeakyReLU}(a_{e}^{T}\cdot [W_{1}h_{i}||W_{2}e_{j}]),\;j\in N_{i}\;$$

where $a_{e}^{T}\in R^{1\times d^{'}}$, are learned, and ‖ denotes vector concatenation that is sum here. These attention scores are normalized across all neighbour edges using softmax function, and the attention function is defined as:

(2) $$\alpha_{\mathrm{ij}}={\mathrm{softmax}}_{j}(\mathrm{es}(h_{i},e_{j}))=\frac{\mathrm{es}(h_{i},e_{j})}{\sum_{j\in N_{i}}\exp(\mathrm{es}(h_{i},e_{j}))}$$

Then, edge attention computes a weighted average of the transformed features of the neighbor edges (followed by a non-linearity σ) as the new representation of *i*, using the normalized attention coefficients:

(3) $${h\prime }_{i}=\sigma (\sum_{j\in N_{i}}\alpha_{\mathrm{ij}}\cdot W_{2}e_{j})$$

Canonical atom and bond features (Li *et al.* 2021) are used for node and edge embedding for all GNNs, including one-hot encoding of 74 atom features and 12 bond features (Supplementary Table S1).

Four modules have a state of existence or absence, the passing message needs to be learned by the module when it exists, otherwise will pass directly when it does not exist. Therefore, 16 models can be generated according to the existence status of the 4 modules, and the sub-module status and meaning of these models can be seen in Table 1. The code is available at https://github.com/heyigacu/BitterGNN.

**Table 1.** The modules and means of 16 models.

Son model	Node attention	Edge attention	Weave	GRU/MPNN	Means
M1	TRUE	TRUE	TRUE	TRUE	HGNN (Hybrid GNN)
M2	TRUE	TRUE	TRUE	FALSE	HGNN without MPNN
M3	TRUE	TRUE	FALSE	TRUE	HGNN without weave
M4	TRUE	TRUE	FALSE	FALSE	node and edge attention
M5	TRUE	FALSE	TRUE	TRUE	HGNN without edge attention
M6	TRUE	FALSE	TRUE	FALSE	weave + GAT
M7	TRUE	FALSE	FALSE	TRUE	GAT + MPNN
M8	TRUE	FALSE	FALSE	FALSE	GAT
M9	FALSE	TRUE	TRUE	TRUE	HGNN without node attention
M10	FALSE	TRUE	TRUE	FALSE	edge attention + weave
M11	FALSE	TRUE	FALSE	TRUE	edge attention + MPNN
M12	FALSE	TRUE	FALSE	FALSE	edge attention
M13	FALSE	FALSE	TRUE	TRUE	weave + MPNN
M14	FALSE	FALSE	TRUE	FALSE	weave
M15	FALSE	FALSE	FALSE	TRUE	MPNN
M16	FALSE	FALSE	FALSE	FALSE	GCN

The 8 public datasets (Table 2) were used to evaluate our 16 GNN models and other 5 novel GNNs (WLN (Morris *et al.* 2018), PAGTN, AttentiveFP, Graph Transformer and GraphSAGE). We evaluated the performance of total 21 models using 5-fold cross-validation under the same parameters (Table S2), and it was repeated 5 times. Assessment metrics for regression and classification are provided in Supplementary Part S1. We selected three metrics of regression (R2, MAE, RMSE) and five metrics of classification (ACC, AP, F1, MCC, AUC) for evaluation. Since different indicators have their own evaluation characteristics, we adopted a comprehensive evaluation method, ranking the model in various measures (ranking in reverse order) and then taking an average value. The higher the score, the better the performance of the model.

**Table 2.** The information of 8 public datasets.

Dataset type	Dataset name	Description	Numbers	Reference
Regression	delaney	ESOL Water solubility	1128	(Delaney, 2004)
	Lipop	Lipop Lipophilicity	4200	(Shen et al., 2021)
	FreeSolv	FreeSolv Solvation free energy	642	(Shen et al., 2021)
	PDBbind	PDBbind-refined	3040	(Shen et al., 2021)
Classification	N6512	Ames mutagenicity data set	6506	(Hansen et al., 2009)
	BBBP	BBBP Blood-brain barrier penetration	2039	(Shen et al., 2021)
	ClinTox2	ClinTox Clinical trial toxicity	1478	(Shen et al., 2021)
	BACE	BACE-1 benchmark set	1513	(Shen et al., 2021)

### 2.3 Comparation of bitterness predictors

To test the performance of bitter predictors based on GNN, we compared them with the other mainstream predictor for predicting bitterness (Fig. 1F). We rewrote the code for CNN and MLP to predict bitter/non-bitter and bitter/sweet using python as much as possible based on the original article (Bo *et al.* 2022). Similarly, we re-implement BitterPredict based on Adapt boost, which requires the descriptor of the molecule generated by schrodinger's QikProp module as input. BitterX and BitterSweetForest are web-based versions that allow bitterness prediction at http://mdl.shsmu.edu.cn/BitterX/ and https://insilico-cyp.charite.de/VirtualTaste/, respectively.

**Figure 2.**
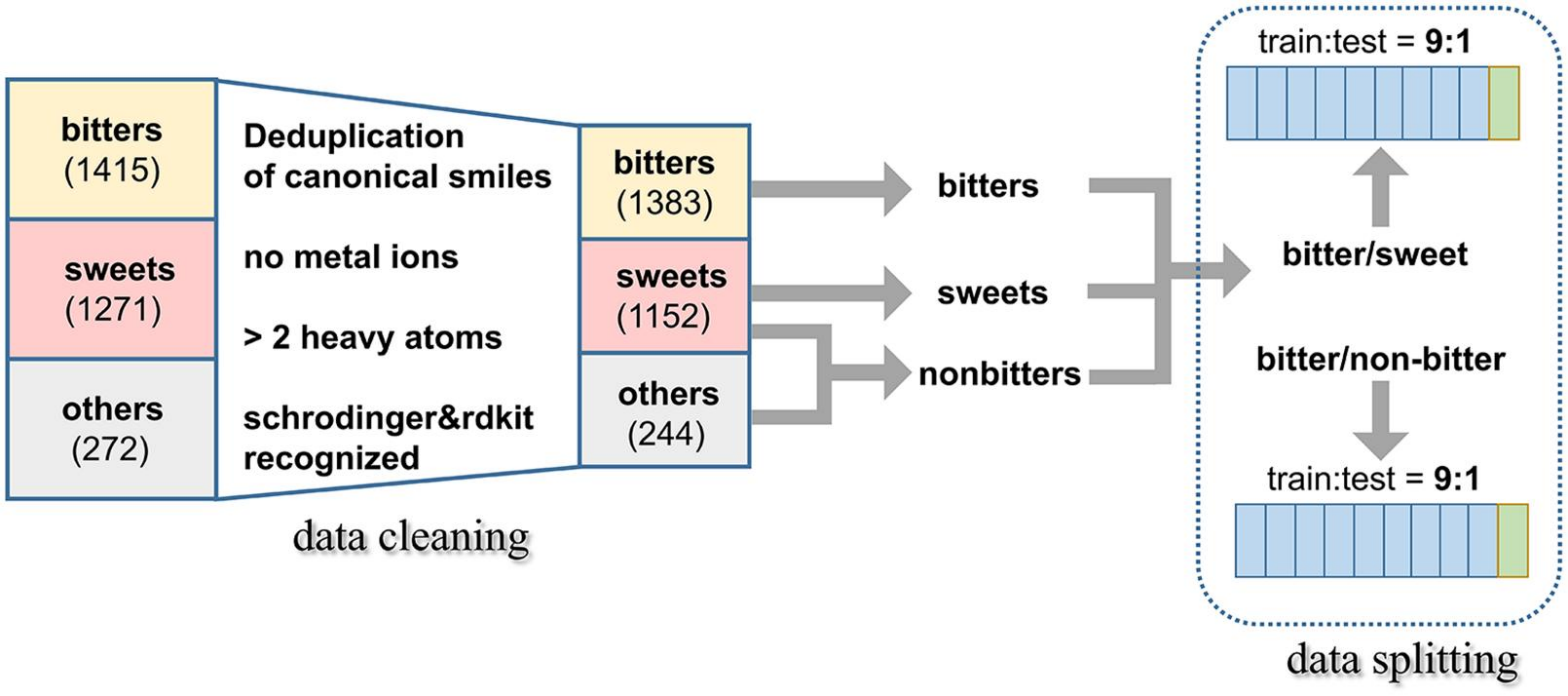
The data cleaning and splitting of bitter dataset.

In order to obtain a common molecular set as input for all comparison methods, we cleaned the original bitter dataset with the filter rules shown in Fig. 2. The detailed data cleaning rules are as below:

1. Molecules are uniformly converted into canonical smiles and deduplicated.
2. Delete molecules containing metal ions.
3. Delete molecules with less than 2 heavy atoms.
4. Delete molecules that schrodinger (https://www.schrodinger.com) and RDKit cannot read.

After data cleaning, we paired bitter/sweet and bitter/non-bitter datasets and divide each data into train set and test set by 9:1 randomly. To prevent contamination of the test set, all hyperparameters are determined by optimizing mean AUC of 5-fold cross-validation in the training set. All the training parameters of GNNs and models are compared are shown in Supplementary Table S2.

### 2.4 Post analysis

Factor analysis was performed using the Python-based Factor_Analysis module. We calculate 195 molecular descriptors (see Supplementary Table S3) for all molecules in the bitter dataset. Bartlett's sphericity test and Kaiser–Meyer–Olkin (KMO) test were performed then on the descriptor features of the bitter/sweet dataset and the bitter/non-bitter dataset, respectively. To ensure smooth matrix decomposition during the calculation, we removed some features, following the rule that no feature should have more than 900 zeros in the numerator.

Next, the number of common factors was determined for the two datasets, and the analysis model was established according to the maximum variance factor rotation, and the variance contribution rate and component matrix are calculated. Finally, the *t*-test was performed for the features whose component matrix value is greater than 0.8.

OpenBabel (O'Boyle *et al.* 2011) was first used to convert all the bitters from SMILES to PDBQT format, and then Vina (Trott and Olson 2010) was used to docking them to the bitter receptor TAS2R46. Next, we carried out the correlation analysis on 195 descriptors with the affinity to bitter receptors, and the Pearson correlation coefficient greater than 0.63 is considered to be a strong correlation.

## 3 Results

### 3.1 Performance of HGNN

**Figure 3.**
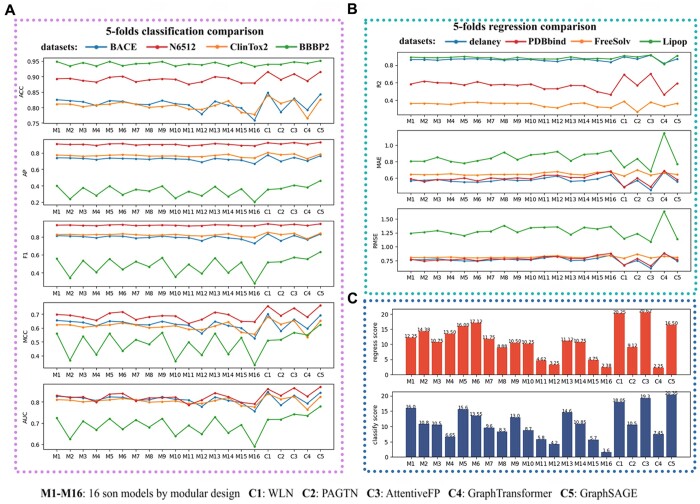
The 21 models test on 8 public datasets. (A) 5 Classification metrics (ACC, AP, F1, MCC, AUC) of the models test on 4 classification datasets, (B) 3 regression metrics (R2, MAE, RMSE) of the models test on 4 regression datasets. (C) Comprehensive regression and classification scores of the models.

As can be seen from the Fig. 3, Model 1 has the best performance (here we use the comprehensive score, which is the average rank of the regression or categorical evaluation indicator on several datasets, and the higher the ranking, the higher the score) among 16 sub-models for classification problems, but not as good as WLN, PAGTN and GraphSAGE. Model 1 integrates weave, node attention, edge attention and MPNN to have stronger learning ability, we called it Hybrid Graph Neural Network (HGNN). According to our design, every 4 models in 16 models is a cycle, and the later the cycle, the simpler the model, but have worse learning ability. Note that C4 model here is just a simple implementation of Graph Transformer, without positional coding and pre-training. We review how HGNN transmits messages from one node to another node. First, the initial node information passed through the linear layer (n2n) and then passed through the node attention layer, and the adjacent edge information was aggregated with the linear layer (e2n) and the edge attention layer, and then the learned node information and adjacent edge information were obtained through the linear layer (un). Finally, it was selectively forgotten or remembered through the GRU layer with the initial node information, and finally passed to the target node. At the same time, the edge information was also updated at this time, and the initial node information after the linear layer (n2e) was added to the edge information after the linear layer (e2n), and then the updated edge is obtained by a linear layer (ue). As for why HGNN achieves better performance than other basic four models, we believe that the combination of models has greater complexity, so that it has stronger robustness in the face of more complex data, which has been confirmed in the ablation experiment composed of these 16 combined models. Finally, we select four GNNs with the best classification performance as the models for bitterness prediction.

### 3.2 Performance of bitter prediction based on GNN

The comparison results of mainstreaming predictors for bitter/non-bitter and bitter/sweet prediction was shown in Table 3. In bitter/non-bitter prediction, AttentiveFP, GraphSAGE and WLN have a good performance (AUC > 0.85) on the external test dataset. BoMLP with RDKFP as input performs best (AUC = 0.86) in the contrast methods. In contrast, HGNN performed less well, with accuracy and AUC values of 0.81 and 0.82, respectively. The worst performer is BitterX, presumably the reason is the insufficient training set of the website version. Traditional machine learning methods like BitterPredict and BoMLP with descriptors as input also perform poorly, potentially because the number of descriptors is too few to extract the critical properties of the molecule and can’t to solve the noise. These demonstrated that GNNs with atom and bond information as node and edge feature embedding are sufficient to identify and classify the key information of the molecule.

**Table 3.** Comparison results of predictors for bitter/non-bitter and bitter/sweet prediction.

Classification Type	Model	Algorithm	Input	TN	FP	FN	TP	TPR	TNR	PRE	ACC	AP	F1	MCC	AUC
Bitter/Non-bitter	BoCNN	CNN	Molecular Figure	132	31	33	82	0.71	0.81	0.73	0.77	0.64	0.72	0.52	0.76
	BoMLP	MLP	Descriptors	119	44	33	82	0.71	0.73	0.65	0.72	0.58	0.68	0.44	0.72
	BoMLP	MLP	RDKFP	141	22	16	99	0.86	0.86	0.82	0.86	0.76	0.84	0.72	0.86
	BitterPredict	Adapt Boost	Descriptors	133	30	32	83	0.72	0.82	0.73	0.78	0.65	0.73	0.54	0.77
	VisualTaste	Random Forest	Descriptors	95	68	20	95	0.83	0.58	0.58	0.68	0.55	0.68	0.41	0.7
	BitterX	SVM	Descriptors	38	125	18	97	0.84	0.23	0.44	0.49	0.43	0.58	0.09	0.54
	HGNN	GNN	Atom and Bond Feature	121	42	12	103	0.9	0.74	0.71	0.81	0.68	0.79	0.63	0.82
	WLN	GNN	Atom and Bond Feature	140	23	14	101	0.88	0.86	0.81	0.87	0.77	0.84	0.73	0.87
	AttentiveFP	GNN	Atom and Bond Feature	150	13	24	91	0.79	0.92	0.88	0.87	0.78	0.83	0.72	0.86
	GraphSAGE	GNN	Atom Feature	146	17	18	97	0.84	0.9	0.85	0.87	0.78	0.85	0.74	0.87
Bitter/Sweet	BoCNN	CNN	Molecular Figure	114	25	29	86	0.75	0.82	0.78	0.79	0.69	0.76	0.57	0.78
	BoMLP	MLP	Descriptors	108	31	37	78	0.68	0.78	0.72	0.73	0.63	0.7	0.46	0.73
	BoMLP	MLP	RDKFP	126	13	23	92	0.8	0.91	0.88	0.86	0.79	0.84	0.71	0.85
	HGNN	GNN	Atom and Bond Feature	122	17	18	97	0.84	0.88	0.85	0.86	0.79	0.85	0.72	0.86
	WLN	GNN	Atom and Bond Feature	122	17	21	94	0.82	0.88	0.85	0.85	0.77	0.83	0.7	0.85
	AttentiveFP	GNN	Atom and Bond Feature	130	9	22	93	0.81	0.94	0.91	0.88	0.82	0.86	0.76	0.87
	GraphSAGE	GNN	Atom Feature	127	12	22	93	0.81	0.91	0.89	0.87	0.8	0.85	0.73	0.86

However, in the bitter/sweet test, the 4 GNN predictors and BoMLP with RDKFP as input maintain similar performance about with value of 0.86. The worst performers were BoCNN and BoMLP with descriptors as input, presumably, the reason is that the image size of the molecule set by BoCNN is too small. In addition, the performance of HGNN in the bitter/sweet dataset is higher than that of the bitter/non-bitter dataset, which is caused by the more inconsistent characteristics of non-bitter data, which indicates that the generalization ability of HGNN needs to be strengthened.

### 3.3 Factor analysis

**Figure 4.**
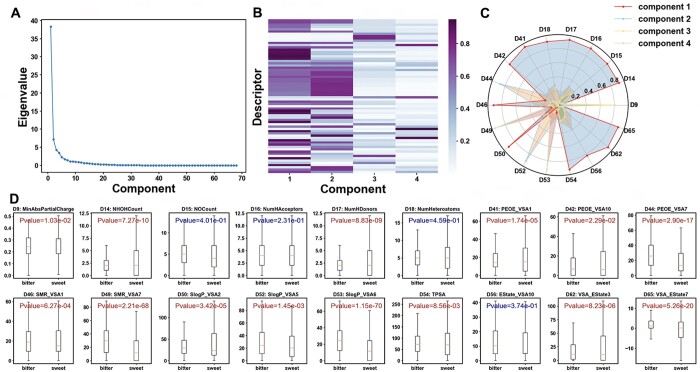
Factor analysis for 68 descriptors of bitter/sweet dataset. (A) Eigenvalues for 68 descriptors of bitter/sweet dataset. (B) Component matrix of first 4 components for the 68 descriptors. (C) Values of the screened 18 descriptors for 4 primary components. (D) T-test of 18 descriptors on the bitter/sweet dataset.

An adequacy test of the bitter/sweet data was conducted, revealing a *P*-value of .00 for Bartlett’s Test and a KMO Test value of 0.92. These results indicate that the data was suitable for factor analysis. The first four common factors accounted for 76.98% of the total variance. The original 68 descriptors were reduced to 18 descriptors after applying a filter on values greater than the threshold of 0.8 in the composition matrix (refer to Fig. 4B). Figure 4C illustrated that the majority of the descriptor variables contributed to the first component, with D44 and D52 contributing to the second component, D9 to the third, and D49 and D53 to the fourth. A T-test on the 18 descriptors of the bitter/sweet dataset, as depicted in Fig. 4D, revealed significant distribution differences among 14 descriptors. These differences were primarily found in hydrogen bonding (NumHDonors and NHOHCount), molecular polarization (SMR_VSA and TPSA), hydrophobic and hydrophilic interactions (SlogP_VSA), and electric charge (PEOE_VSA, EState_VSA9, and MinAbsPartialCharge). Hence, molecules with smaller interacting surface areas, such as those with charge interactions and polarity interactions, may have been more likely to elicit a sweet taste. Most notable is the indicators of lipophilicity, SLogP, which suggested that bitter molecules may have stronger hydrophobic interactions than sweet molecules (Di Pizio *et al.* 2019).

**Figure 5.**
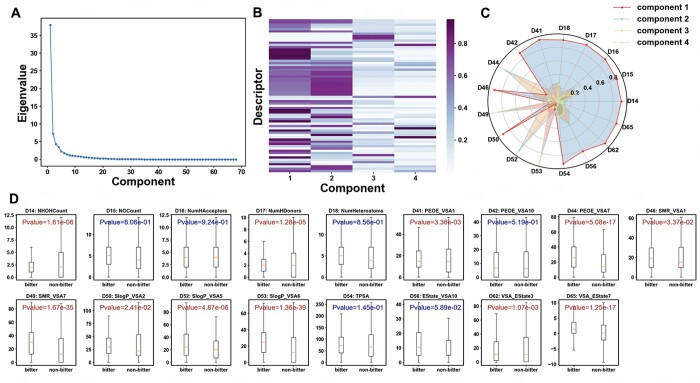
Factor analysis for 68 descriptors of bitter/non-bitter dataset. (A) Eigenvalues for 68 descriptors of bitter/non-bitter dataset. (B) Component matrix of first 4 components for the 68 descriptors. (C) Values of the screened 17 descriptors for 4 primary components. (D) T-test of the 17 descriptors on the bitter/non-bitter dataset.

The bitter/non-bitter dataset resulted in a *P*-value of .92 for Bartlett’s Test and a KMO Test value of 10 × 10^−8^, indicating that this dataset was also suitable for factor analysis. As depicted in Fig. 5A, the selection of four common factors for subsequent factor analysis was considered appropriate, with these factors accounting for 76.55% of the total variance. A threshold of 0.8 was applied to screen the 68 descriptors in the component matrix (Fig. 5B), which resulted in 17 descriptors passing the threshold. The majority of these descriptors contributed to the first component, with the exceptions of D44 and D52 contributing to the second component, and D49 and D53 contributing to the fourth. A T-test on these descriptors revealed that only 11 descriptors showed significant differences. This number was lower than that of the bitter/non-bitter dataset, which could potentially be attributed to the broader distribution of the non-bitter dataset.

**Figure 6.**
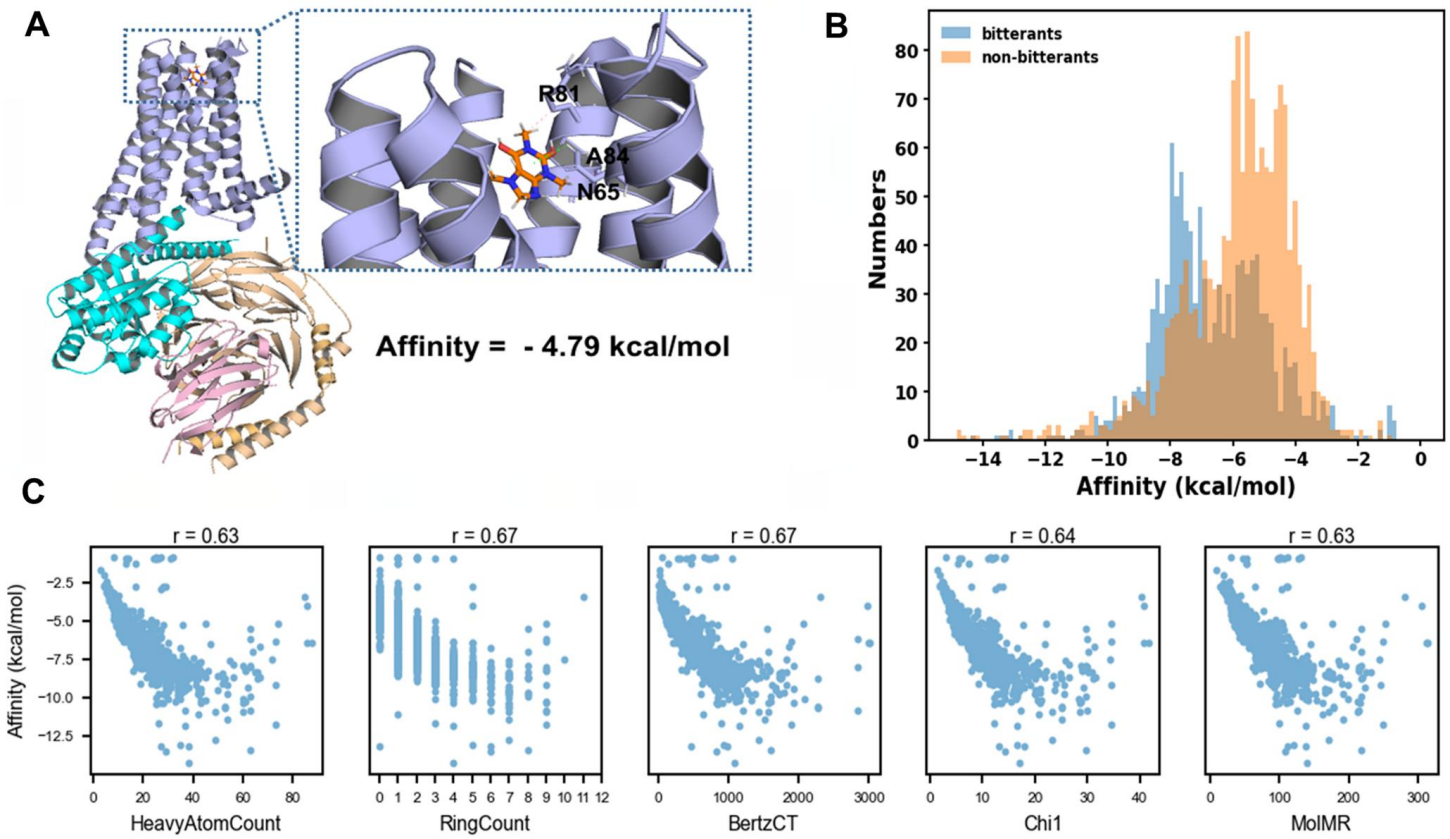
(A) The molecular docking of caffeine with TAS2R46. (B) The distribution difference of the affinity to TAS2R46 of bitterants and sweeteners respectively. (C) Pearson correlation coefficient between count of heavy atoms, count of rings, BertzCT, Chi1 and molecular relative mass with Affinity to TAS2R46.

Figure 6A displayed the molecular docking result of the well-known bitterant caffeine (Poole and Tordoff 2017) and the broad-spectrum bitter receptor TAS2R46 (Xu *et al.* 2022). The molecular docking results of bitter and non-bitter substances with TAS2R46, as determined by Autodock Vina, revealed that TAS2R46’s affinity for these substances does not follow a normal distribution (Fig. 6B). The P-value of 2.91e-44 from the Mann-Whitney test indicated that the affinity of bitter agents for TAS2R46 is significantly higher than that of non-bitter agents (*P* < .05), which is in accordance with our cognitive expectations. Further correlation analysis of the affinity of bitterant descriptors (Fig. 6C) revealed that the count of heavy atoms, count of rings, BertzCT, Chi1, and molecular relative mass had a strong positive correlation with TAS2R46 affinity. Consequently, molecules with a larger mass, more complex shape, and valence electronic information may exhibit higher affinity to TAS2R46. We hypothesize that molecules with a larger mass and greater complexity are potentially more toxic, and that the evolution of the bitter taste receptor in human functions to deter the ingestion of such substances.

## 4 Discussion

**Figure 7.**
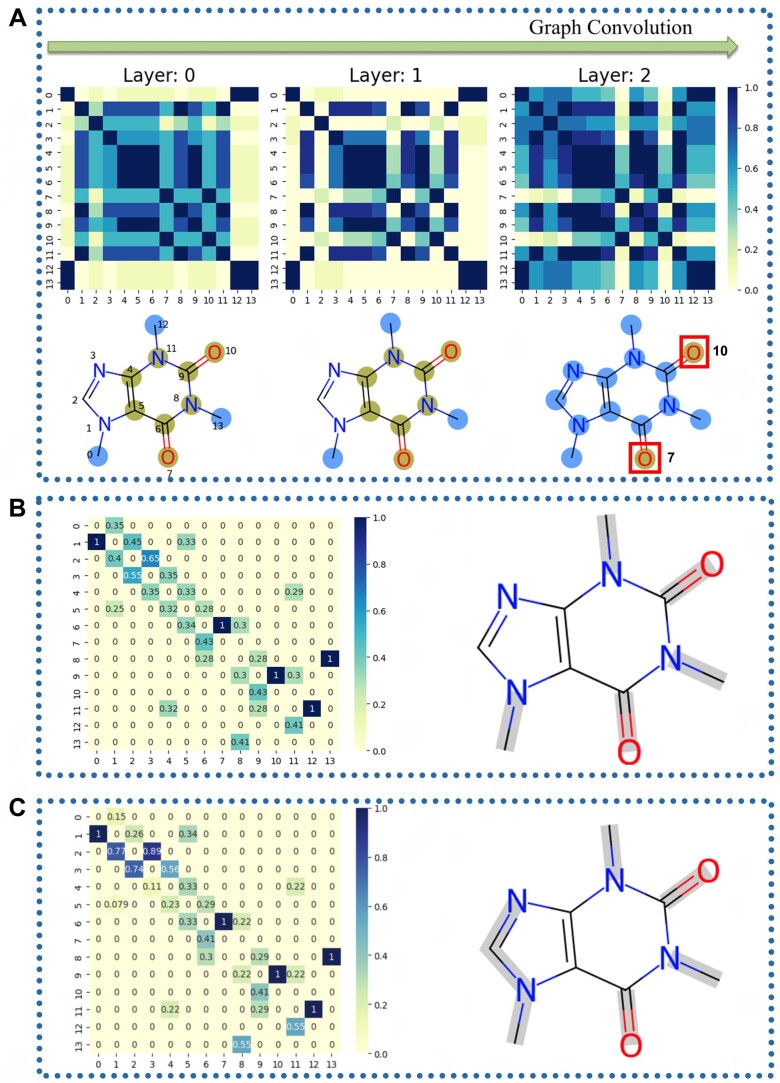
(A) As the number of layers of the graph convolution deepens, HGNN learned the correlation matrix of the atomic characteristics of the caffeine molecule, The caffeine molecule shows two sets of atoms with an internal correlation of > 0.3, represented in green and blue, respectively. (B) Node attention and (C) Edge attention in the last layer of HGNN when caffeine as input to predict the bitterness, bonds with an attention value > 0.7 are marked in ray.

Graph neural networks hold significant advantages in molecular property prediction and classification. To obtain the interpretability of the HGNN, we explore the mechanism of the model learning molecular features. The probability that caffeine is predicted as bitter by HGNN is 96%, so we explored how HGNN learns bitterness characteristics from the molecular structure of caffeine. As shown in Fig. 7A, the initial nodes embedding characteristics of the caffeine molecule can be divided into two groups (one group is atoms 0, 12 and 13, and the other is atoms 4, 5, 6 and 9) based on correlation. When at the first layer of graph convolutions, we only find that atoms 2, 7 and 10 have a decrease in correlation with other atoms, but when the graph convolutions at the second layer, we find that the atoms except atom 7 and 10 show a strong connection, and these two oxygen atoms are almost cut off from them, so we think that these two carbonyl oxygen atoms play an important role in bitterness of caffeine. In addition, we also explored the attention mechanism of HGNN. We output the last layer of attention coefficient (Fig. 7B) of HGNN's node attention when caffeine is used as input, and we find that the value of the attention coefficient matrix of all neighbor atoms is not completely symmetrical, which indicates that the attention from neighbors (horizontal axis) and the attention to neighbors (vertical axis) are not consistent, and the atom with the highest attention by neighbors is atom 6 N. In edge attention (Fig. 7C), more edges, such as N1-C2 and C2 = N3, are recognized by attention mechanisms.

**Figure 8.**
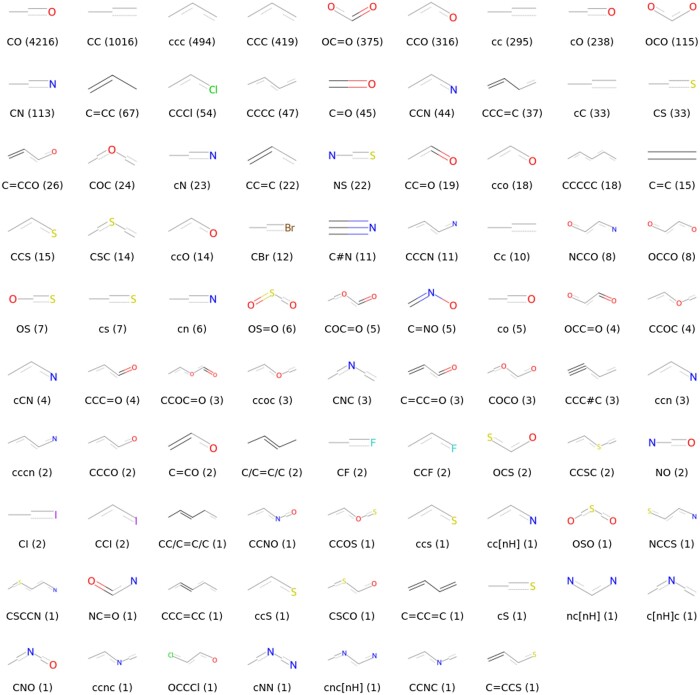
88 key substructures searched from bitter molecules, below each substructure is the substructure SMILES and the number of occurrences.

In order to explore the bitterness-related chemical groups that HGNN learned from more bitter molecules other than caffeine, we used a depth-first search (DFS) algorithm to traverse the molecular graph to search the continuous path combined with atomic pairs with HGNN attention score > 0.5, which we call the continuous attention substructure, and they may play an important role in the bitterness recognition of molecules by HGNN. A total of 8370 substructures were excavated from 1383 bitter molecules, and 88 key substructures were obtained after dropping duplication (Fig. 8), of which more than half were hydroxyl carbon atoms, which indicated that hydroxyl groups accounted for an important degree in the attention of carbon atoms.

Building on this novel approach to taste prediction, the taste of natural product molecules from COCONUT (https://coconut.naturalproducts.net/) has been successfully predicted. After removing 351 of these error molecules, 103 762 of the remaining 406 919 molecules were predicted to be bitter and 303 157 were predicted to be non-bitter. To understand the scale of this achievement, consider that an experienced taster evaluating 100 substances per day would require nearly eleven years to complete this task, not accounting for the difficulty of acquiring molecules and potential toxicity of the molecules. Hence, this model marks a significant advancement in the field, accelerating the process of predicting tastes and serving as a valuable tool for both taste research and flavor development.

By using the simple, modularized model HGNN which can compete with the latest GNN models, this research offers fresh insights into how straightforward algorithms and architectures can tackle complex tasks. The way the modules work together demonstrates the potential of modular designs to yield results greater than the sum of their parts, shows a cumulative effect where 1 + 1 > 2, inviting further exploration for novel applications of established algorithms. It provides a novel way to design deep learning models.

For the first time, this modular design and the latest GNN models have been applied to the prediction of bitter-sweet tastes, achieving an accuracy of 0.86, which is almost on par with the highest level model in this field with an accuracy of 0.88. This innovative approach propels the advancement of the taste prediction domain and represents a significant contribution to our understanding of this intricate biological system.

The TastePD website, developed alongside the model, currently hosts a bitter-sweet taste predictor and a complementary database. Efforts are underway to expand this into a multi-flavor predictor, aiming to provide a comprehensive tool for taste prediction research. Welcome to continue to follow our progress. This development will greatly enhance accessibility and applicability of taste prediction models, deepening our comprehensive understanding of taste.

Regarding practical considerations such as individual variances in taste perception thresholds, the relationship between flavor intensity and concentration, and the dynamic nature of datasets in real-world settings, it is hopeful that with an increase in training data, the performance of the taste prediction model will continue to improve, further contributing to the exploration of the fascinating world of taste.

## 5 Conclusion

Through a novel modular design method, we obtained a new graph neural network HGNN, which is superior to the traditional GNN like weave, GAT, and MPNN. Based on HGNN and other three well-performed GNNs, BitterGNNs were developed, which have the same performance than even better than the best-known method RDKFP-MLP. And through factor analysis and correlation analysis, we determined that molecules with greater interacting surface areas, mass, shape complexity are associated with bitterness. In addition, we developed a server TastePD where the models and datasets were deployed.

## Supplementary Material

vbae041_Supplementary_Data

## References

[vbae041-B1] Banerjee P , Preissner R. BitterSweetForest: a random Forest based binary classifier to predict bitterness and sweetness of chemical compounds. Front Chem 2018;6:93. 29696137 10.3389/fchem.2018.00093 PMC5905275

[vbae041-B2] Bo W , Qin D, Zheng X et al Prediction of bitterant and sweetener using structure-taste relationship models based on an artificial neural network. Food Res Int 2022;153:110974. 35227485 10.1016/j.foodres.2022.110974

[vbae041-B3] Brody S, Alon U, Yahav E. How attentive are graph attention networks? ICLR 2022.

[vbae041-B4] Charoenkwan P , Nantasenamat C, Hasan MM et al BERT4Bitter: a bidirectional encoder representations from transformers (BERT)-based model for improving the prediction of bitter peptides. Bioinformatics 2021;37:2556–62. 33638635 10.1093/bioinformatics/btab133

[vbae041-B5] Chen B, Barzilay R, Jaakkola TS. Path-augmented graph transformer network. ICML 2019.

[vbae041-B6] Dagan-Wiener A , Nissim I, Ben Abu N et al Bitter or not? BitterPredict, a tool for predicting taste from chemical structure. Sci Rep 2017;7:12074. 28935887 10.1038/s41598-017-12359-7 PMC5608695

[vbae041-B7] Delaney JS. ESOL: estimating aqueous solubility directly from molecular structure. J Chem Inf Comput Sci 2004;44:1000–5. 15154768 10.1021/ci034243x

[vbae041-B8] Di Pizio A , Ben Shoshan-Galeczki Y, Hayes JE et al Bitter and sweet tasting molecules: it's complicated. Neurosci Lett 2019;700:56–63. 29679682 10.1016/j.neulet.2018.04.027

[vbae041-B9] Drewnowski A , Gomez-Carneros C. Bitter taste, phytonutrients, and the consumer: a review. Am J Clin Nutr 2000;72:1424–35. 11101467 10.1093/ajcn/72.6.1424

[vbae041-B10] Duvenaud DK et al Convolutional networks on graphs for learning molecular fingerprints. NIPS 2015;2:2224–32.

[vbae041-B11] Freund Y , Schapire RE. A decision-theoretic generalization of on-line learning and an application to boosting. J Comput Syst Sci 1997;55:119–39.

[vbae041-B12] Gilmer J, Schoenholz SS, Riley PF et al Neural message passing for quantum chemistry. ICML 2017.

[vbae041-B13] Hamilton WL , Ying Z, Leskovec J. Inductive representation learning on large graphs. NIPS, 2017;1025–1035.

[vbae041-B14] Hansen K , Mika S, Schroeter T et al Benchmark data set for in silico prediction of ames mutagenicity. J Chem Inf Model 2009;49:2077–81. 19702240 10.1021/ci900161g

[vbae041-B15] Hu W, Liu B, Gomes J et al Strategies for pre-training graph neural networks. ICLR2020a.

[vbae041-B16] Hu W, Fey M, Zitnik M et al Open graph benchmark: datasets for machine learning on graphs. NIPS2020b;33:22118–33.

[vbae041-B17] Huang W , Shen Q, Su X et al BitterX: a tool for understanding bitter taste in humans. Sci Rep 2016;6:23450. 27040075 10.1038/srep23450 PMC4819188

[vbae041-B18] Kearnes S , McCloskey K, Berndl M et al Molecular graph convolutions: moving beyond fingerprints. J Comput Aided Mol Des 2016;30:595–608. 27558503 10.1007/s10822-016-9938-8 PMC5028207

[vbae041-B19] Kipf T , Welling M. Semi-Supervised Classification with Graph Convolutional Networks. ICLR 2017.

[vbae041-B20] Li M , Zhou J, Hu J et al DGL-LifeSci: an Open-Source toolkit for deep learning on graphs in life science. ACS Omega 2021;6:27233–8. 34693143 10.1021/acsomega.1c04017 PMC8529678

[vbae041-B21] Lu C, Liu Q, Wang C et al Molecular property prediction: a multilevel quantum interactions modeling perspective. AAAI 2019;33:1052–60.

[vbae041-B22] Mennella JA , Spector AC, Reed DR et al The bad taste of medicines: overview of basic research on bitter taste. Clin Ther 2013;35:1225–46. 23886820 10.1016/j.clinthera.2013.06.007 PMC3772669

[vbae041-B23] Morris C , Ritzert M, Fey M et al Weisfeiler and leman go neural: higher-order graph neural networks. AAAI 2018;33:4602–9.

[vbae041-B24] O'Boyle NM , Banck M, James CA et al Open babel: an open chemical toolbox. J Cheminform 2011;3:33. 21982300 10.1186/1758-2946-3-33 PMC3198950

[vbae041-B25] Poole RL , Tordoff MG. The taste of caffeine. J Caffeine Res 2017;7:39–52. 28660093 10.1089/jcr.2016.0030 PMC5488350

[vbae041-B26] Schütt K, Kindermans PJ, Sauceda HE et al SchNet: A continuous-filter convolutional neural network for modeling quantum interactions. NIPS 2017;992–1002.

[vbae041-B27] Shen WX , Zeng X, Zhu F et al Out-of-the-box deep learning prediction of pharmaceutical properties by broadly learned knowledge-based molecular representations. Nat Mach Intell 2021;3:334–43.

[vbae041-B28] Trott O , Olson AJ. AutoDock vina: improving the speed and accuracy of docking with a new scoring function, efficient optimization, and multithreading. J Comput Chem 2010;31:455–61. 19499576 10.1002/jcc.21334 PMC3041641

[vbae041-B29] Veličković P. Everything is connected: graph neural networks. Curr Opin Struct Biol 2023;79:102538. 36764042 10.1016/j.sbi.2023.102538

[vbae041-B30] Wang Y , Hu J, Lai J et al TF3P: three-Dimensional force fields fingerprint learned by deep capsular network. J Chem Inf Model 2020;60:2754–65. 32392062 10.1021/acs.jcim.0c00005

[vbae041-B31] Witt M. Anatomy and development of the human taste system. Handb Clin Neurol 2019;164:147–71. 31604544 10.1016/B978-0-444-63855-7.00010-1

[vbae041-B32] Wooding SP , Ramirez VA, Behrens M et al Bitter taste receptors: genes, evolution and health. Evol Med Public Health 2021;9:431–47. 35154779 10.1093/emph/eoab031 PMC8830313

[vbae041-B33] Xiong Z , Wang D, Liu X et al Pushing the boundaries of molecular representation for drug discovery with the graph attention mechanism. J Med Chem 2020;63:8749–60. 31408336 10.1021/acs.jmedchem.9b00959

[vbae041-B34] Xu K, Hu W, Leskovec J, Jegelka S. How powerful are graph neural networks? ICLR 2019.

[vbae041-B35] Xu W , Wu L, Liu S et al Structural basis for strychnine activation of human bitter taste receptor TAS2R46. Science 2022;377:1298–304. 36108005 10.1126/science.abo1633

