# Supplementary material for: Prediction of bitterness based on modular designed graph neural network
Source: Bioinform Adv. 2024 Mar 13;4(1):vbae041. doi: 10.1093/bioadv/vbae041 (PMC10987211; doi:10.1093/bioadv/vbae041)
Supplement: vbae041_Supplementary_Data [file vbae041_supplementary_data.docx]

## Supplementary Materials

## Prediction of bitterness based on modular

## designed graph neural network

Yi He, Kaifeng Liu, Yuyang Liu, Weiwei Han^*^

Affiliation: Key Laboratory for Molecular Enzymology and Engineering of Ministry of Education, School of Life Science, Jilin University, 2699 Qianjin Street, Changchun 130012, China

* Correspondence: +86 13756550860 (W.H.)

E-mail: weiweihan@jlu.edu.cn (W.H.)

#### Part I **Metrics**

**1.1 Metrics for Regression Performance**

$$MAE=\frac{1}{n}\sum_{i=1}^{n} \left| y_{i}-\hat{y_{i}} \right|$$

MAE: Mean Absolute Error, which measures the average absolute difference between predicted and actual values.

$$MSE=\frac{1}{n}\sum_{i=1}^{n} \left( y_{i}-\hat{y_{i}} \right)^{2}$$

MSE: Mean Squared Error, a metric that calculates the average of the squares of the differences between predicted and actual values, emphasizing the impact of larger errors.

$$MAPE=\frac{1}{n}\sum_{i=1}^{n} \left| \frac{y_{i}-\hat{y_{i}}}{y_{i}} \right|\times100$$

MAPE: Mean Absolute Percentage Error, a measure that calculates the average of the absolute percentage differences between predicted and actual values, providing a relative error metric that is scale-independent.

$$RMSE=\sqrt{\frac{1}{n}\sum_{i=1}^{n} \left( y_{i}-\hat{y_{i}} \right)^{2}}$$

RMSE: Root Mean Squared Error, a metric that computes the square root of the average of the squared differences between predicted and actual values, providing a sense of the magnitude of the error while maintaining the same units as the quantity being estimated.

1.2 Metrics for Classification Performance

$$Accuracy=\frac{TP+TF}{TP+TF+FP+FN}$$

Accuracy: A metric that calculates the proportion of correct predictions (both positive and negative) over all predictions, providing an overall measure of the model’s performance.

$$Precision=\frac{TP}{TP+FP}$$

Precision: Also known as Positive Predictive Value, a metric that measures the proportion of true positives among all positive predictions, evaluating the model’s ability to correctly identify positive cases.

$$Sensitivity=Recall=\frac{TP}{TP+FN}$$

Sensitivity: Also known as Recall or True Positive Rate, a metric that calculates the proportion of actual positives that are correctly identified as such, assessing the model’s ability to correctly identify all relevant instances.

$$Specificity=\frac{TN}{TP+FN}$$

Specificity: Also known as True Negative Rate, a metric that measures the proportion of actual negatives that are correctly identified as such, evaluating the model’s ability to correctly identify all non-relevant instances.

$$F1=\frac{2*Precision*Recall}{Precision+Recall}=\frac{2*TP}{2*TP+FP+FN}$$

F1 Score: A harmonic mean of precision and recall, providing a balance between the two metrics, especially useful when dealing with imbalanced datasets.

$$AP=\frac{1}{n}\sum_{k=1}^{n} P\left( k \right)\cdot rel\left( k \right)$$

AP: Average Precision, a metric that averages the precision values at different recall levels, providing an overall measure of a model’s precision-recall performance across all possible classification thresholds.

$$MCC=\frac{TP\times TN-FP\times FN}{\sqrt{\left( TP+FP \right)\left( TP+FN \right)\left( TN+FP \right)\left( TN+FN \right)}}$$

MCC: Matthews Correlation Coefficient, a metric that measures the quality of binary classifications, providing a balanced measure even when classes are of very different sizes. It takes into account true and false positives and negatives and is generally regarded as a balanced measure which can be used even if the classes are of very different sizes.

#### Part II Supplementary Tables

| Table S1. Canonical atom feature and bond feature | | | |
| --- | --- | --- | --- |
| Atom Feature | Counter | Bond Feature | Counter |
| C', 'N', 'O', 'S', 'F', 'Si', 'P', 'Cl', 'Br', 'Mg', 'Na', 'Ca', 'Fe', 'As', 'Al', 'I', 'B', 'V', 'K', 'Tl', 'Yb', 'Sb', 'Sn', 'Ag', 'Pd', 'Co', 'Se', 'Ti', 'Zn', 'H', 'Li', 'Ge', 'Cu', 'Au', 'Ni', 'Cd', 'In', 'Mn', 'Zr', 'Cr', 'Pt', 'Hg', 'Pb' | 43 | Bond Type | 4 |
| Degree | 11 | Is Conjugated | 1 |
| Implicit Valence | 7 | Is In Ring | 1 |
| Formal Charge | 1 | Stereo | 6 |
| Number of Radical Electrons | 1 |  |  |
| HybridizationType | 5 |  |  |
| Is Aromatic | 1 |  |  |
| Total Number of Hs | 5 |  |  |

| Table S2. Basic Parameters of the 21 models | |
| --- | --- |
| Model | Parameters |
| M1 | node_gat = True, edge_gat = True, weave = True, mpnn = True, n_node_feats=n_node_feats, n_edge_feats=n_edge_feats, num_layers=2, n_heads=5, n_hidden_feats=100, activation=F.relu, attn_activation=nn.LeakyReLU(negative_slope=0.2), attn_dropout=0, feat_dropout=0, xavier_normal=False, optim.Adam(), lr=0.001 |
| M2 | node_gat = True, edge_gat = True, weave = True, mpnn = False, n_node_feats=n_node_feats, n_edge_feats=n_edge_feats, num_layers=2, n_heads=5, n_hidden_feats=100, activation=F.relu, attn_activation=nn.LeakyReLU(negative_slope=0.2), attn_dropout=0, feat_dropout=0, xavier_normal=False, optim.Adam(), lr=0.001 |
| M3 | node_gat = True, edge_gat = True, weave = False, mpnn = True, n_node_feats=n_node_feats, n_edge_feats=n_edge_feats, num_layers=2, n_heads=5, n_hidden_feats=100, activation=F.relu, attn_activation=nn.LeakyReLU(negative_slope=0.2), attn_dropout=0, feat_dropout=0, xavier_normal=False, optim.Adam(), lr=0.001 |
| M4 | node_gat = True, edge_gat = True, weave = False, mpnn = False, n_node_feats=n_node_feats, n_edge_feats=n_edge_feats, num_layers=2, n_heads=5, n_hidden_feats=100, activation=F.relu, attn_activation=nn.LeakyReLU(negative_slope=0.2), attn_dropout=0, feat_dropout=0, xavier_normal=False, optim.Adam(), lr=0.001 |
| M5 | node_gat = True, edge_gat = False, weave = True, mpnn = True, n_node_feats=n_node_feats, n_edge_feats=n_edge_feats, num_layers=2, n_heads=5, n_hidden_feats=100, activation=F.relu, attn_activation=nn.LeakyReLU(negative_slope=0.2), attn_dropout=0, feat_dropout=0, xavier_normal=False, optim.Adam(), lr=0.001 |
| M6 | node_gat = True, edge_gat = False, weave = True, mpnn = False, n_node_feats=n_node_feats, n_edge_feats=n_edge_feats, num_layers=2, n_heads=5, n_hidden_feats=100, activation=F.relu, attn_activation=nn.LeakyReLU(negative_slope=0.2), attn_dropout=0, feat_dropout=0, xavier_normal=False, optim.Adam(), lr=0.001 |
| M7 | node_gat = True, edge_gat = False, weave = False, mpnn = True, n_node_feats=n_node_feats, n_edge_feats=n_edge_feats, num_layers=2, n_heads=5, n_hidden_feats=100, activation=F.relu, attn_activation=nn.LeakyReLU(negative_slope=0.2), attn_dropout=0, feat_dropout=0, xavier_normal=False, optim.Adam(), lr=0.001 |
| M8 | node_gat = True, edge_gat = False, weave = False, mpnn = False, n_node_feats=n_node_feats, n_edge_feats=n_edge_feats, num_layers=2, n_heads=5, n_hidden_feats=100, activation=F.relu, attn_activation=nn.LeakyReLU(negative_slope=0.2), attn_dropout=0, feat_dropout=0, xavier_normal=False, optim.Adam(), lr=0.001 |
| M9 | node_gat = False, edge_gat = True, weave = True, mpnn = True, n_node_feats=n_node_feats, n_edge_feats=n_edge_feats, num_layers=2, n_heads=5, n_hidden_feats=100, activation=F.relu, attn_activation=nn.LeakyReLU(negative_slope=0.2), attn_dropout=0, feat_dropout=0, xavier_normal=False, optim.Adam(), lr=0.001 |
| M10 | node_gat = False, edge_gat = True, weave = True, mpnn = False, n_node_feats=n_node_feats, n_edge_feats=n_edge_feats, num_layers=2, n_heads=5, n_hidden_feats=100, activation=F.relu, attn_activation=nn.LeakyReLU(negative_slope=0.2), attn_dropout=0, feat_dropout=0, xavier_normal=False, optim.Adam(), lr=0.001 |
| M11 | node_gat = FalseFalse, edge_gat = True, weave = False, mpnn = True, n_node_feats=n_node_feats, n_edge_feats=n_edge_feats, num_layers=2, n_heads=5, n_hidden_feats=100, activation=F.relu, attn_activation=nn.LeakyReLU(negative_slope=0.2), attn_dropout=0, feat_dropout=0, xavier_normal=False, optim.Adam(), lr=0.001 |
| M12 | node_gat = False, edge_gat = True, weave = False, mpnn = False, n_node_feats=n_node_feats, n_edge_feats=n_edge_feats, num_layers=2, n_heads=5, n_hidden_feats=100, activation=F.relu, attn_activation=nn.LeakyReLU(negative_slope=0.2), attn_dropout=0, feat_dropout=0, xavier_normal=False, optim.Adam(), lr=0.001 |
| M13 | node_gat = False, edge_gat = False, weave = True, mpnn = True, n_node_feats=n_node_feats, n_edge_feats=n_edge_feats, num_layers=2, n_heads=5, n_hidden_feats=100, activation=F.relu, attn_activation=nn.LeakyReLU(negative_slope=0.2), attn_dropout=0, feat_dropout=0, xavier_normal=False, optim.Adam(), lr=0.001 |
| M14 | node_gat = False, edge_gat = False, weave = False, mpnn = True, n_node_feats=n_node_feats, n_edge_feats=n_edge_feats, num_layers=2, n_heads=5, n_hidden_feats=100, activation=F.relu, attn_activation=nn.LeakyReLU(negative_slope=0.2), attn_dropout=0, feat_dropout=0, xavier_normal=False, optim.Adam(), lr=0.001 |
| M15 | node_gat = False, edge_gat = False, weave = False, mpnn = True, n_node_feats=n_node_feats, n_edge_feats=n_edge_feats, num_layers=2, n_heads=5, n_hidden_feats=100, activation=F.relu, attn_activation=nn.LeakyReLU(negative_slope=0.2), attn_dropout=0, feat_dropout=0, xavier_normal=False, optim.Adam(), lr=0.001 |
| M16 | node_gat = False, edge_gat = False, weave = False, mpnn = False, n_node_feats=n_node_feats, n_edge_feats=n_edge_feats, num_layers=2, n_heads=5, n_hidden_feats=100, activation=F.relu, attn_activation=nn.LeakyReLU(negative_slope=0.2), attn_dropout=0, feat_dropout=0, xavier_normal=False, optim.Adam(), lr=0.001 |
| C1 | node_in_feats=74, edge_in_feats=12, node_out_feats=100, n_layers=2, project_in_feats=True, set_comparison=True, optim.Adam(), lr=0.001 |
| C2 | node_in_feats=74, node_hid_feats=100, node_out_feats=100, edge_feats=12, depth=2, nheads=5, dropout=0.1, activation=nn.LeakyReLU(0.2), mode='sum', optim.Adam(), lr=0.001 |
| C3 | node_feat_size=74,edge_feat_size=12,num_layers=2,num_timesteps=2,graph_feat_size=100,dropout=0., optim.Adam(), lr=0.001 |
| C4 | n_dim=74, hidden_dim=100, out_dim=100, num_layers=2, num_heads=5, optim.Adam(), lr=0.001 |
| C5 | in_feats=74, hidden_feats=[100,100], activation=None, dropout=None, aggregator_type=None, optim.Adam(), lr=0.001 |

M1-16 mean combined GNN models and C1-5 mean contrast new GNN models. node_gat mean node-node attention, edge_gat mean node-edge attention, weave mean add edge learning module, mpnn add a GRU module, n_node_feats mean number of node feature, n_edge_feats mean number of edge feature, num_layers mean number of convolution layers, n_heads mean attention headers, n_hidden_feats mean number of hidden feature, activation mean activation function, attn_activation mean attention activation function, attn_dropout mean attention dropout, feat_dropout mean dropout in hidden layers, xavier_normal mean if add xavier normalization, optim.Adam() mean use Adam optimizer, lr mean learning rate.

| Table S3. The information of 195 descriptors | | |
| --- | --- | --- |
| No. | Descriptors | Meaning |
| 1 | MolWt | molecular weight |
| 2 | ExactMolWt | exact molecular weight |
| 3-5 | FpDensityMorgan1-3 | morgan fingerprint |
| 6 | HeavyAtomMolWt | average molecular weight of the molecule ignoring hydrogens |
| 7 | MaxAbsPartialCharge | maximum absolute partial charge |
| 8 | MaxPartialCharge | maximum partial charge |
| 9 | MinAbsPartialCharge | minimal absolute partial charge |
| 10 | MinPartialCharge | minimal partial charge |
| 11 | NumRadicalElectrons | the number of radical electrons |
| 12 | NumValenceElectrons | the number of valence electrons |
| 13 | FractionCSP3 | the fraction of C atoms that are SP3 hybridized |
| 14 | HeavyAtomCount | the number of heavy atoms |
| 15 | NHOHCount | the number of NHs or OHs |
| 16 | NOCount | the number of Nitrogens and Oxygens |
| 17 | NumAliphaticCarbocycles | the number of aliphatic (containing at least one non-aromatic bond) carbocycles |
| 18 | NumAliphaticHeterocycles | the number of aliphatic (containing at least one non-aromatic bond) heterocycles |
| 19 | NumAliphaticRings | the number of aliphatic (containing at least one non-aromatic bond) rings |
| 20 | NumAromaticCarbocycles | the number of aromatic carbocycles |
| 21 | NumAromaticHeterocycles | the number of aromatic heterocycles |
| 22 | NumAromaticRings | the number of aromatic rings |
| 23 | NumHAcceptors | the number of Hydrogen Bond Acceptors |
| 24 | NumHDonors | the number of Hydrogen Bond Donors |
| 25 | NumHeteroatoms | the number of Heteroatoms |
| 26 | NumRotatableBonds | the number of Rotatable Bonds |
| 27 | NumSaturatedCarbocycles | the number of saturated carbocycles |
| 28 | NumSaturatedHeterocycles | the number of saturated heterocycles |
| 29 | NumSaturatedRings | the number of saturated rings |
| 30 | RingCount | the number of rings |
| 31 | BalabanJ | a float containing the J value |
| 32 | Chi0 | Atomic connectivity index (order 0) |
| 33 | BertzCT | a topological index meant to quantify “complexity” of molecules |
| 34 | Chi0n | the average of the net charges of all atoms in a molecule |
| 35 | Chi0v | the average of the ability of all atoms in a molecule to absorb electrons |
| 36 | Chi1 | Atomic connectivity index (order 1) |
| 37 | Chi1n | the average of the sum of the net charges of all first-order adjacent atomic pairs in a molecule |
| 38 | Chi1v | the average of the sum of the electron-absorbing abilities of all first-order adjacent atomic pairs in the molecule |
| 39 | Chi2n | the average of the sum of the net charges of all second-order adjacent atomic pairs in the molecule |
| 40 | Chi2v | the average of the sum of the electron-absorbing abilities of all second-order neighboring atomic pairs in the molecule |
| 41 | Chi3n | the average of the sum of the net charges of all three-order neighboring atomic pairs in the molecule |
| 42 | Chi3v | the average of the sum of the electron-absorbing abilities of all tertiary adjacent atomic pairs in the molecule |
| 43 | Chi4n | the average of the sum of the net charges of all four-order adjacent atomic pairs in the molecule |
| 44 | Chi4v | the average of the sum of the electron-absorbing abilities of all four-order adjacent atomic pairs in the molecule |
| 45 | HallKierAlpha | the polarity and charge distribution of the molecule |
| 46 | Ipc | the information content of the coefficients of the characteristic polynomial of the adjacency matrix of a hydrogen-suppressed graph of a molecule |
| 47-49 | Kappa1-3 | molecular surface area is calculated to describe properties such as solubility and biodistribution of molecules |
| 50 | LabuteASA | the electron cloud density distribution based on molecules describes the degree of bending and three-dimensional shape of molecules |
| 51-64 | PEOE_VSA1-14 | sum of the van der Waals surface area (Å2) where the partial charge of atom i (qi) is in a range, intended to capture direct electrostatic interactions. |
| 65-74 | SMR_VSA1-10 | sum of the van der Waals surface area where Molar Refractivity for atom i in a range, intended to capture polarizability |
| 75-86 | SlogP_VSA1-12 | sum of the van der Waals surface area where Log of the octanol/water partition coefficient (including implicit hydrogens) for atom i in a range, intended to capture hydrophobic and hydrophilic effects |
| 87 | TPSA | topological polar surface area: the total surface area of polar molecules within a compound |
| 88 | pyLabuteASA | Labute’s Approximate Surface Area |
| 89-99 | EState_VSA1-11 | sum of the van der Waals surface area where electrotopological state index for atom i in a range |
| 100-109 | VSA_EState1-10 | sum of the electrotopological state index where evan der Waals surface are for atom i in a range |
| 110 | MolMR | Wildman-Crippen MR value: Molar refractive index |
| 111 | MolLogP | Wildman-Crippen LogP value: Lipid-water partition coefficient |
| 112 | fr_Al_COO | the number of aliphatic carboxylic acids |
| 113 | fr_Al_OH | the number of aliphatic hydroxyl groups |
| 114 | fr_Al_OH_noTert | the number of aliphatic hydroxyl groups excluding tert-OH |
| 115 | fr_ArN | the number of N functional groups attached to aromatics |
| 116 | fr_Ar_COO | the number of Aromatic carboxylic acide |
| 117 | fr_Ar_N | the number of aromatic nitrogens |
| 118 | fr_Ar_NH | the number of aromatic amines |
| 119 | fr_Ar_OH | the number of aromatic hydroxyl groups |
| 120 | fr_COO | the number of carboxylic acids |
| 121 | fr_COO2 | the number of carboxylic acids |
| 122 | fr_C_O | the number of carbonyl O |
| 123 | fr_C_O_noCOO | the number of carbonyl O, excluding COOH |
| 124 | fr_C_S | the number of thiocarbonyl |
| 125 | fr_HOCCN | the number of C(OH)CCN-Ctert-alkyl or C(OH)CCNcyclic |
| 126 | fr_Imine | the number of Imines |
| 127 | fr_NH0 | the number of Tertiary amines |
| 128 | fr_NH1 | the number of Secondary amines |
| 129 | fr_NH2 | the number of Primary amines |
| 130 | fr_N_O | the number of hydroxylamine groups |
| 131 | fr_Ndealkylation2 | the number of tert-alicyclic amines (no heteroatoms, not quinine-like bridged N) |
| 132 | fr_Nhpyrrole | the number of H-pyrrole nitrogens |
| 133 | fr_SH | the number of thiol groups |
| 134 | fr_aldehyde | the number of aldehydes |
| 135 | fr_alkyl_carbamate | the number of alkyl carbamates (subject to hydrolysis) |
| 136 | fr_alkyl_halide | the number of alkyl halides |
| 137 | fr_allylic_oxid | the number of allylic oxidation sites excluding steroid dienone |
| 138 | fr_amide | the number of amides |
| 139 | fr_amidine | the number of amidine groups |
| 140 | fr_aniline | the number of anilines |
| 141 | fr_aryl_methyl | the number of aryl methyl sites for hydroxylation |
| 142 | fr_azide | the number of azide groups |
| 143 | fr_azo | the number of azo groups |
| 144 | fr_barbitur | the number of barbiturate groups |
| 145 | fr_benzene | the number of benzene rings |
| 146 | fr_benzodiazepine | the number of benzodiazepines with no additional fused rings |
| 147 | fr_bicyclic | the number of Bicyclic |
| 148 | fr_diazo | the number of diazo groups |
| 149 | fr_dihydropyridine | the number of dihydropyridines |
| 150 | fr_epoxide | the number of epoxide rings |
| 151 | fr_ester | the number of esters |
| 152 | fr_ether | the number of ether oxygens (including phenoxy) |
| 153 | fr_furan | the number of furan rings |
| 154 | fr_guanido | the number of guanidine groups |
| 155 | fr_halogen | the number of halogens |
| 156 | fr_hdrzine | the number of hydrazine groups |
| 157 | fr_hdrzone | the number of hydrazone groups |
| 158 | fr_imidazole | the number of imidazole rings |
| 159 | fr_imide | the number of imide groups |
| 160 | fr_isocyan | the number of isocyanates |
| 161 | fr_isothiocyan | the number of isothiocyanates |
| 162 | fr_ketone | the number of ketones |
| 163 | fr_ketone_Topliss | the number of ketones excluding diaryl, a,b-unsat |
| 164 | fr_lactam | the number of beta lactams |
| 165 | fr_lactone | the number of cyclic esters (lactones) |
| 166 | fr_methoxy | the number of methoxy groups -OCH3 |
| 167 | fr_morpholine | the number of morpholine rings |
| 168 | fr_nitrile | the number of nitriles |
| 169 | fr_nitro | the number of nitro groups |
| 170 | fr_nitro_arom | the number of nitro benzene ring substituent |
| 171 | fr_nitro_arom_nonortho | the number of non-ortho nitro benzene ring substituents |
| 172 | fr_nitroso | the number of nitroso groups, excluding NO2 |
| 173 | fr_oxazole | the number of oxazole rings |
| 174 | fr_oxime | the number of oxime groups |
| 175 | fr_para_hydroxylation | the number of para-hydroxylation sites |
| 176 | fr_phenol | the number of phenols |
| 177 | fr_phenol_noOrthoHbond | the number of phenolic OH excluding ortho intramolecular Hbond substituents |
| 178 | fr_phos_acid | the number of phosphoric acid groups |
| 179 | fr_phos_ester | the number of phosphoric ester groups |
| 180 | fr_piperdine | the number of piperdine rings |
| 181 | fr_piperzine | the number of piperzine rings |
| 182 | fr_priamide | the number of primary amides |
| 183 | fr_prisulfonamd | the number of primary sulfonamides |
| 184 | fr_pyridine | the number of pyridine rings |
| 185 | fr_quatN | the number of quarternary nitrogens |
| 186 | fr_sulfide | the number of thioether |
| 187 | fr_sulfonamd | the number of sulfonamides |
| 188 | fr_sulfone | the number of sulfone groups |
| 189 | fr_term_acetylene | the number of terminal acetylenes |
| 190 | fr_tetrazole | the number of tetrazole rings |
| 191 | fr_thiazole | the number of tetrazole rings |
| 192 | fr_thiocyan | the number of thiocyanates |
| 193 | fr_thiophene | the number of thiophene rings |
| 194 | fr_unbrch_alkane | the number of unbranched alkanes of at least 4 members (excludes halogenated alkanes) |
| 195 | fr_urea | the number of urea groups |
